# Seronegative immunity to SARS-CoV-2: a case study

**DOI:** 10.1186/s13223-022-00715-w

**Published:** 2022-08-30

**Authors:** Kyle D. Miller, Amie M. Ashcraft, Courtney S. Pilkerton, Carl D. Shrader

**Affiliations:** Department of Family Medicine, Medical Center Drive, West Virginia University, Box 9152, Morgantown, WV 26506 USA

**Keywords:** SARS-CoV-2, Coronavirus, COVID-19, Immunity

## Abstract

**Background:**

COVID-19 presents with a variable clinical course from asymptomatic to severe respiratory distress with nearly 25% mortality in mechanically-ventilated patients. As such, there is uncertainty regarding how host factors modulate the disease course.

**Case Presentation:**

This report examines these factors in two geriatric patients with multiple comorbid conditions who were residents of the long-term care facility in West Virginia that was the epicenter of COVID-19 in the state. Each patient had substantial, unprotected exposure to SARS-CoV-2 with subsequent negative PCR and antibody testing.

**Conclusions:**

These cases could represent an important step in understanding host factors that modulate the disease course and susceptibility of patients exposed to SARS-CoV-2, and illustrate the need for further research into host resistance relating to this pandemic.

## Background

At the time of the writing of this report, SARS-CoV-2, the virus responsible for COVID-19, has spread to more than 90 million people globally, with over 23 million cases originating in the United States (US) alone [1]. Though physical distancing guidelines, stay-at-home orders, and face shielding in public spaces have had a demonstrable impact on SARS-CoV-2 transmission in the US and elsewhere, the death toll continues to climb and there remains an urgent search for both treatments and preventative measures. However, when a pathogen presents with such a variable clinical course—from asymptomatic to severe respiratory distress with nearly 25% mortality in mechanically-ventilated patients—there is uncertainty regarding how host factors are modulating the disease course [2]. This report examines such factors from the perspective of a long-term care facility (LTCF) in West Virginia that was the epicenter of COVID-19 in the state. Two cases are detailed in which each patient had substantial, unprotected exposure to SARS-CoV-2 with subsequent negative PCR and antibody testing, suggesting host resistance that has not been previously well-documented.

## Case presentation

### Case 1

Patient X is an 80-year-old Caucasian male with an extensive past medical history including late-stage Alzheimer’s dementia, peripheral vascular disease, hypertension, hyperlipidemia, diabetes mellitus type 2, hypothyroidism, atherosclerotic coronary artery disease, heart failure and chronic kidney disease. Of note, Patient X’s wife is a resident of the same nursing home and shares a room with him. His wife tested positive in the initial swabbing of the facility following sentinel case detection, along with 21 other residents and 13 staff members.

At the onset of the outbreak, the facility implemented strict facility-wide infection control measures, including full personal protective equipment (PPE) consisting of N95 mask, contact gown, gloves, and emphasized hand hygiene. The 1st floor east-wing of the nursing home was transitioned into the COVID-19 isolation area, and all negative patients were relocated to other units. However, Patient X was allowed to continue to reside with his wife in the same room both for the sake of compassionate care and under the assumption that he was likely an asymptomatic carrier whose first PCR swab returned a false negative. Due to the patient’s dementia and cognitive communication deficits, he was unable to follow any of the suggested face-masking or hand hygiene guidelines. For instance, several staff members observed him helping feed his wife with his fingers without any hand hygiene before or after. These contacts occurred before, during, and after his wife’s symptomatic period, thus suggesting an exceedingly high likelihood of exposure to SARS-CoV-2.

After detection of the initial case of SARS-CoV-2 infection, the LTCF implemented weekly PCR testing to contain the outbreak. Patient X had negative PCR nasopharyngeal tests over a 3-month period. It was postulated that X might have had the infection prior to sentinel case detection on 03/22/2020 and was therefore demonstrating PCR negativity secondary to preexisting humoral immunity. Serology testing was performed, and patient X returned negative for both IgG and IgM antibodies.

### Case 2

Patient Y is a 71-year-old Caucasian female with a past medical history including Parkinson’s disease, major depressive disorder, osteoporosis, type 2 diabetes, peripheral vascular disease, hypertension, hyperlipidemia, history of DVT, and lumbosacral spondylosis. Like Patient X, Patient Y was a long-term resident of the LTCF who resided in the building throughout the entirety of the COVID-19 outbreak. Notably, the patient’s first PCR test on 03/23/20 with the initial sweep of the nursing home was unable to be processed. Because she could not be ruled out for SARS-CoV-2, she was kept in the isolation unit to prevent any further transmission to confirmed negative residents. As the isolation unit began to reach capacity, a positive patient was subsequently roomed with Patient Y on 03/26/20. This roommate was symptomatic starting on 03/23/20 with a fever of 102.3F documented that evening. She progressed to become lethargic with rapid, labored breathing, hypoxemia, and cough. Ultimately this resident was transferred out of the facility on 04/06/2020 and expired on 04/07/2020. Prior to this patient’s discharge from the facility and while roomed with Patient Y, PPE could not be maintained due to the patient’s declining clinical status. However, despite Patient Y’s exposure to a symptomatic roommate, she subsequently tested negative via PCR over a 3-month period, with negative IgG and IgM testing as well.

## Discussion and conclusions

The authors believe that the above patient cases represent the first observed and documented, unprotected exposures of high-risk patients in a controlled environment, paired with concurrent PCR and serologic testing that failed to demonstrate a readily identifiable reason for immunity. Bang et. al detailed several principles of host coronavirus resistance in the 1970’s, which provides a relevant framework for today’s pandemic [3–5]. These principles of resistance included expression of specific cellular receptors modulating viral entry, host factors and genetics that modulate intracellular viral replication, and humoral/cellular defenses that target viral particles. Cell surface angiotensin-converting enzyme 2 (ACE-2) and the protease TMPRSS2 appear to be the primary host receptors for SARS-CoV-2 cell entry. Grifoni et al. have published results suggesting that 40–60% of unexposed individuals may have lymphocytes bearing cross-reactivity from common-cold strains of coronaviruses to SARS-CoV-2, which may offer a source to the above patient’s immunity [6]. Genetic immunity at the intracellular level has been demonstrated through host macrophage factors in studies of mice infected with the coronaviruses MHV-2 and MHV-3 (mouse hepatitis virus). However, intracellular immunity has not yet been investigated regarding SARS-CoV-2***.*** Finally, humoral/cellular immunity is being investigated in the forms of vaccination and convalescent plasma donation [7].

An additional consideration would be the viral load and subsequent purported transmissibility of COVID-19 for the originally infected roommate of the above patients. While viral loads unfortunately cannot be obtained retrospectively, the authors can anecdotally report that in all other roommate pairings in the facility in which one roommate tested positive for COVID-19, the second roommate also tested positive with the next round of testing.

At present, the literature regarding possible pharmacologic treatments of COVID-19 is rapidly evolving. Patients have been treated with hydroxychloroquine and azithromycin since early in the pandemic, however, data remain conflicting over the use of these medications and a potential for serious adverse reactions exists in the form of cardiac dysrhythmias [8]. Similarly, several preliminary studies of remdesivir in COVID-19 bear inconclusive results [9]. However, while mortality benefit has not been clearly demonstrated, it has been associated with a reduced time to recovery. Stemming from the purported role of cytokine storming in severe cases, IL-6 modulators such as tocilizumab have been proposed as adjunctive therapies. More recent data do suggest a mortality benefit, with an all-cause mortality reduction associated with a relative risk of 0.89 [10]. However, larger scale studies are still pending, and limited availability alongside prohibitive costs, as well as risk for secondary infection, may become barriers to expanded use [11]. Investigation into glucocorticoid use—particularly dexamethasone—has become a topic of interest relating to COVID-19. A preliminary study published in July 2020 showed a reduction in 28-day mortality among patients receiving low-dose dexamethasone, particularly in the subgroup of patients requiring oxygen support (both invasive and noninvasive) [12]. Several other trials looked at dexamethasone but were stopped after finding similar results to that of the aforementioned preliminary trial. Monoclonal antibody treatments have been granted a recent emergency use authorization, but data at this time points toward a recovery benefit in outpatient cases. Finally, baricitinib is currently under investigation as well, which early data from an unpublished trial suggest a mortality benefit both independently and used in conjunction with dexamethasone [13].

Thus, in this constellation of treatments under investigation, inconsistent and variable transmission reduction measures, and the unknown surrounding immunity post-infection, there is a need to address other approaches to preventing transmission and mortality. While it is unknown from where the host resistance presented in this case report is derived, documentation of such resistance in COVID-19 is an important step in refining our understanding of SARS-CoV-2, and integral in developing future therapeutic strategies.

The above case descriptions represent the first known documentation of host resistance to SARS-CoV-2 despite exposure and confirmed lack of immunoglobulin-derived immunity. Both cases were patients who represent the common characterization of a high-risk COVID-19 patient—geriatric with multiple comorbid conditions and unable to participate in transmission reduction measures. As such, we suggest that these cases could represent an important step in understanding host factors that modulate the disease course and susceptibility of patients exposed to SARS-CoV-2. Furthermore, these cases illustrate the continued need for further research into host resistance relating to this pandemic.

## Data Availability

The datasets during and/or analyzed during the current study available from the corresponding author on reasonable request.

## Abbreviations

SARS-CoV-2: Severe acute respiratory syndrome coronavirus 2
COVID-19: Coronavirus disease of 2019
US: United States
LTCF: Long-term care facility
PCR: Polymerase chain reaction test
PPE: Personal protective equipment
IgG: Immunoglobulin G
IgM: Immunoglobulin M
DVT: Deep vein thrombosis
ACE-2: Angiotensin-converting enzyme 2
TMPRSS2: Transmembrane protease serine 2
MHV-2: Mouse hepatitis virus type 2
MHV-3: Mouse hepatitis virus type 3

## References

[1] Centers for disease control and prevention. coronavirus disease 2019 (COVID-19) in the U.S. 2020. https://covid.cdc.gov/covid-data-tracker/#cases_totalcases. Accessed 15 Sept 2020.

[2] Richardson S, Hirsch JS, Narasimhan M (2020). Presenting characteristics, comorbidities, and outcomes among 5700 patients hospitalized With COVID-19 in the New York city area. JAMA.

[3] Kantoch M, Warwick A, Bang FB (1963). The cellular nature of genetic susceptibility to a virus. J Exp Med.

[4] Shif I, Bang FB (1970). In vitro interaction of mouse hepatitis virus and macrophages from genetically resistant mice. I. Adsorption of virus and growth curves. J Exp Med.

[5] Weiser WY, Bang FB (1977). Blocking of in vitro and in vivo susceptibility to mouse hepatitis virus. J Exp Med.

[6] Grifoni A, Weiskopf D, Ramirez SI, Mateus J, Dan JM, Moderbacher CR, Rawlings SA, Sutherland A, Premkumar L, Jadi RS, Marrama D, de Silva AM, Frazier A, Carlin AF, Greenbaum JA, Peters B, Krammer F, Smith DM, Crotty S, Sette A (2020). Targets of T Cell responses to SARS-CoV-2 coronavirus in humans with COVID-19 disease and unexposed individuals. Cell.

[7] Li L, Zhang W, Hu Y (2020). Effect of convalescent plasma therapy on time to clinical improvement in patients with severe and life-threatening COVID-19: a randomized clinical trial. JAMA.

[8] US FDA. Coronavirus (COVID-19) update: FDA revokes emergency use authorization for chloroquine and hydroxychloroquine. 2020. https://www.fda.gov/news-events/press-announcements/coronavirus-covid-19-update-fda-revokes-emergency-use-authorization-chloroquine-and Accessed 16 June 2020

[9] Beigel JH, Tomashek KM, Dodd LE (2020). Remdesivir for the treatment of Covid-19—preliminary report. N Engl J Med.

[10] Ghosn L, Chaimani A, Evrenoglou T, Davidson M, Graña C, Schmucker C, Bollig C, Henschke N, Sguassero Y, Nejstgaard CH, Menon S, Nguyen TV, Ferrand G, Kapp P, Riveros C, Ávila C, Devane D, Meerpohl JJ, Rada G, Hróbjartsson A, Grasselli G, Tovey D, Ravaud P, Boutron I (2021). Interleukin-6 blocking agents for treating COVID-19: a living systematic review. Cochrane Database Syst Rev.

[11] Klopfenstein T, Zayet S, Lohse A (2020). Tocilizumab therapy reduced intensive care unit admissions and/or mortality in COVID-19 patients. Med Mal Infect.

[12] Horby P, Lim WS, RECOVERY Collaborative Group (2020). Dexamethasone in hospitalized patients with Covid-19—preliminary report. N Engl J Med.

[13] Marconi VC, Ramanan AV, de Bono S, et al. Baricitinib plus standard of care for hospitalized adults with COVID-19. UNPUBLISHED. https://www.medrxiv.org/content/10.1101/2021.04.30.21255934v1.

